# Supplementary material for: Mitochondrial citrate metabolism and efflux regulate BeWo differentiation
Source: Sci Rep. 2023 May 6;13:7387. doi: 10.1038/s41598-023-34435-x (PMC10164164; doi:10.1038/s41598-023-34435-x)

**Mitochondrial citrate metabolism and efflux regulate BeWo differentiation**

**Supplement Figure Legends:**

**Supplemental Figure 1: Confirmation of biochemical and morphologic differentiation**

**in BeWo cells**

A) Schematic of cytotrophoblast fusion into multinucleated syncytiotrophoblasts (Figure created in Biorender) B) qPCR analysis showing upregulation of *CGA, CBG* and *ERVW* gene expression in response to differentiation, n=4; C) Representative images demonstrating morphologic changes associated with forskolin treatment. Blue depicts DAPI nuclear staining and green demonstrates wheat germ agglutinin staining of cell membrane. D) % Fusion=((number of nuclei in syncytia/total number of nuclei)*100%). n=3. Data are representative of mean +/-SEM. ***, p<0.001; ****, p<0.0001.

**Supplemental Figure 2: Uncropped blots from Figure 2C**

Uncropped blots from figure 2C.

**Supplemental Figure 3: Expression of SLC25A1, ACSS2, and ACLY in Trophoblast Stem Cell model of differentiation**

qPCR analysis showing relative expression of A) *CGA,* B) *CGB2,* C) *ERVW-1,* D) *SLC25A1,* E) *ACSS2* and F) *ACLY* mRNA in Trophoblast Stem Cell Model (CT-29). n= 6 biological replicates

SR=self-renewing trophoblast stem cell. ST=syncytiotrophoblast. Data are representative of mean +/-SEM. ***, *p*<0.001 and ****, *p*<0.0001.

**Supplemental Figure 4: Uncropped blots from Figure 3A**

Uncropped blots from Figure 3A

**Supplemental Figure 5: Loss of CIC does not impair morphologic syncytialization**

Representative images demonstrating cell morphology following treatment of BeWo control or CIC Knockout cells with A) DMSO or B) 40 µM forskolin. Blue depicts DAPI nuclear staining, green demonstrates E-cadherin, and magenta represents HCG. Scale bar represents 50 µm.

C) Quantification of Fusion. % Fusion=((number of nuclei in syncytia/total number of nuclei)*100%). n=6.

**Supplemental Figure 6: Uncropped blots from Figure 5**

Uncropped blots from Figure 5A

Supplemental Figure 1:


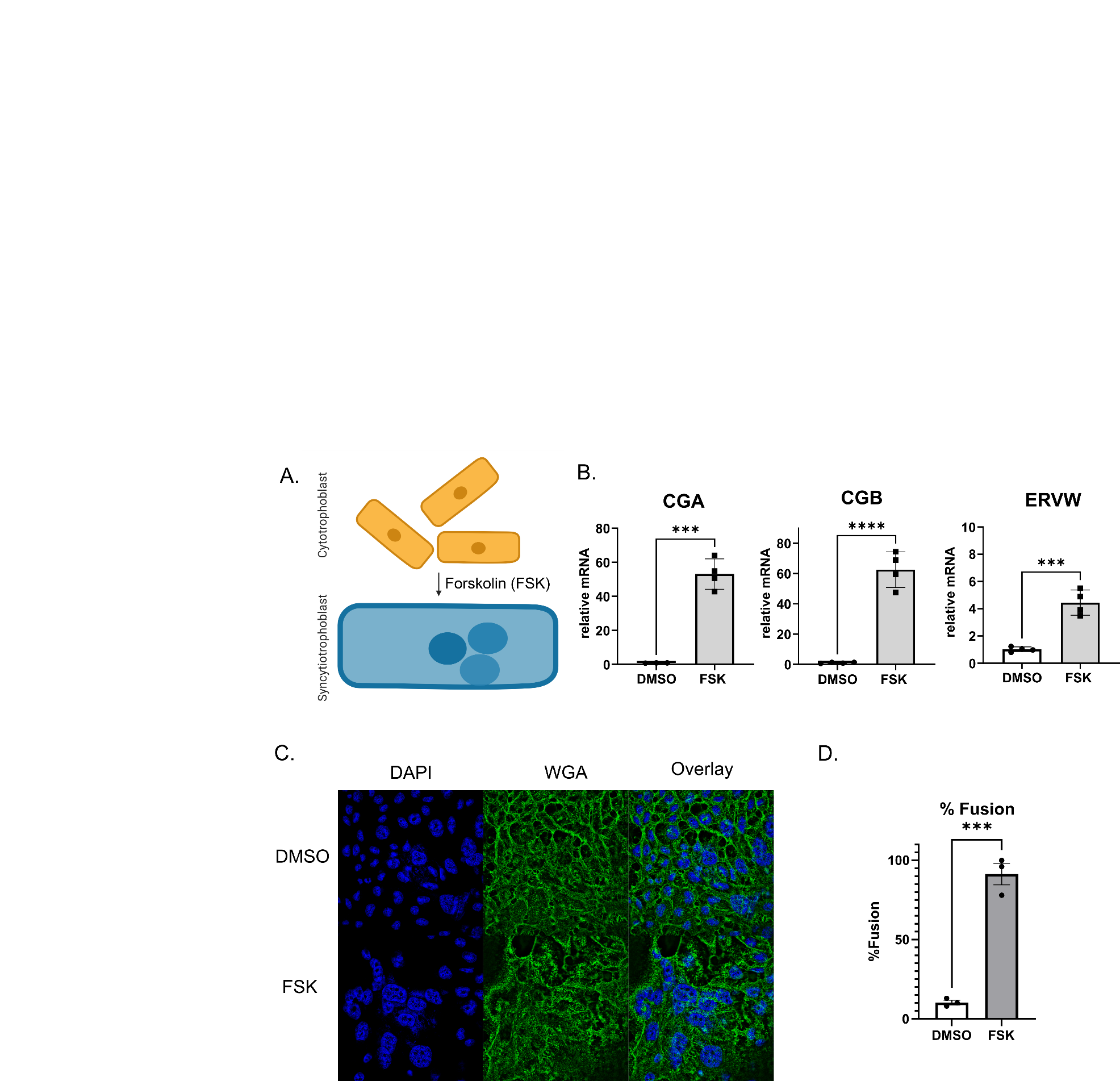


Supplemental Figure 2:


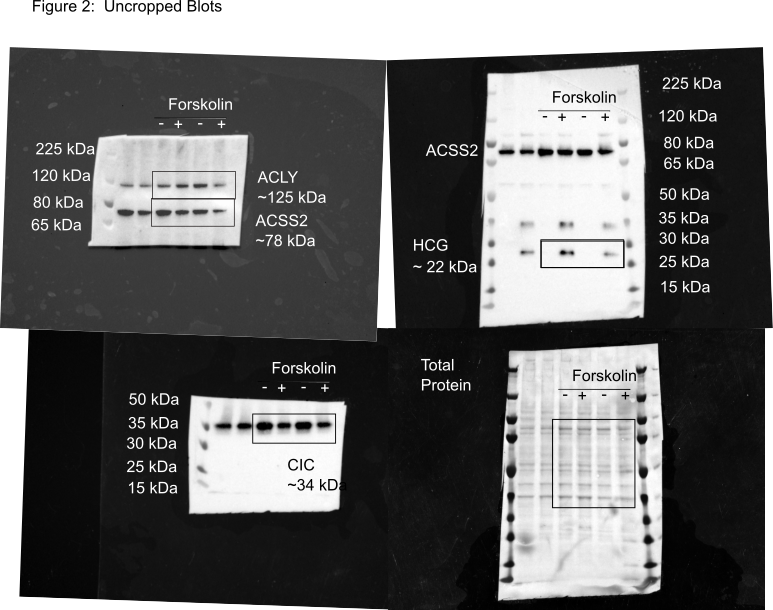


Supplemental Figure 3


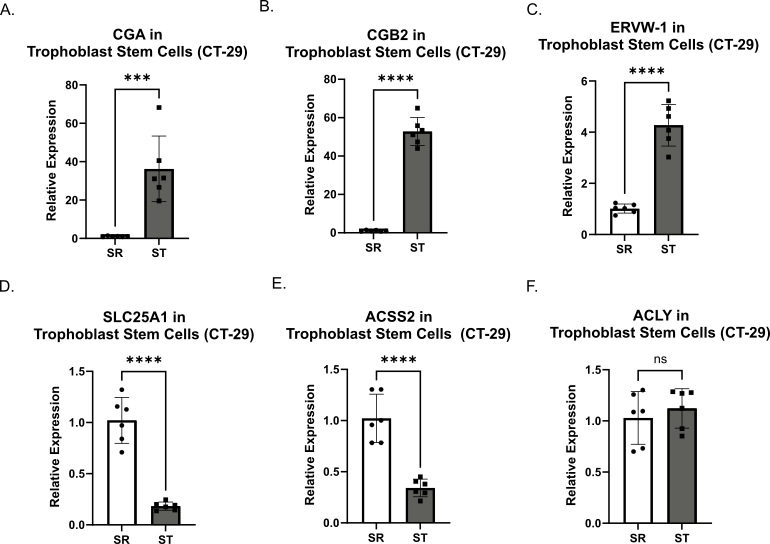


Supplemental Figure 4


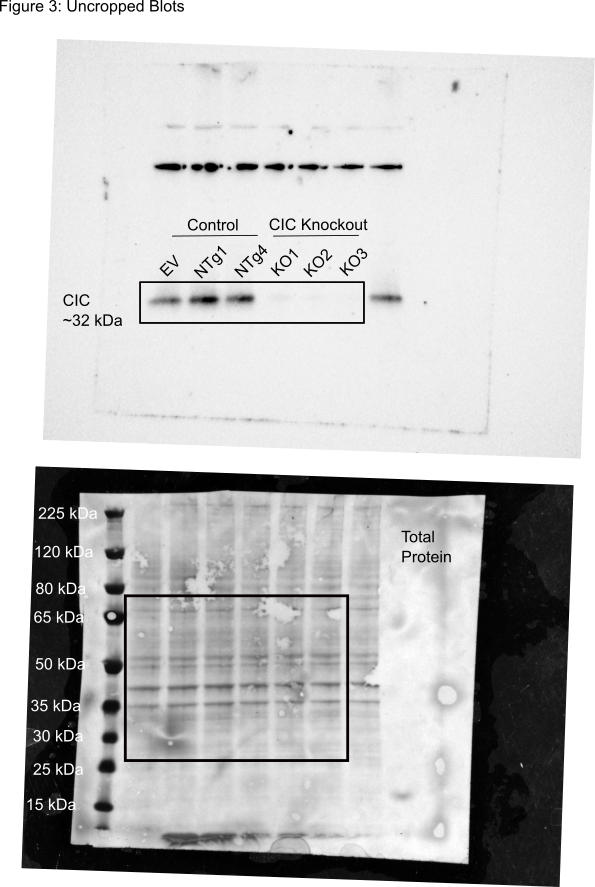


Supplemental Figure 5:


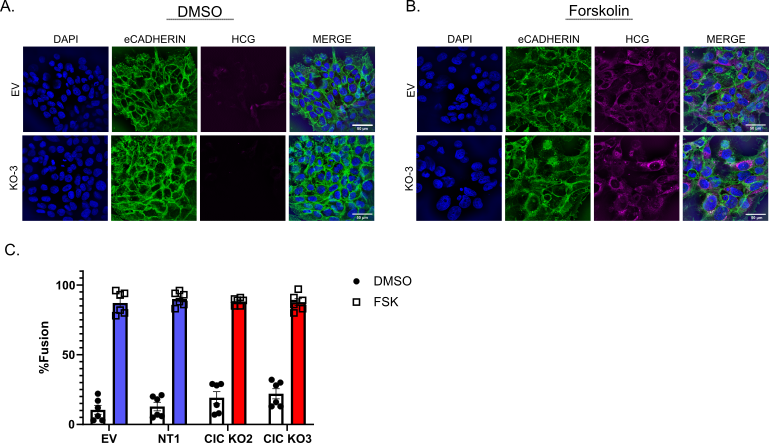


Supplemental Figure 6:
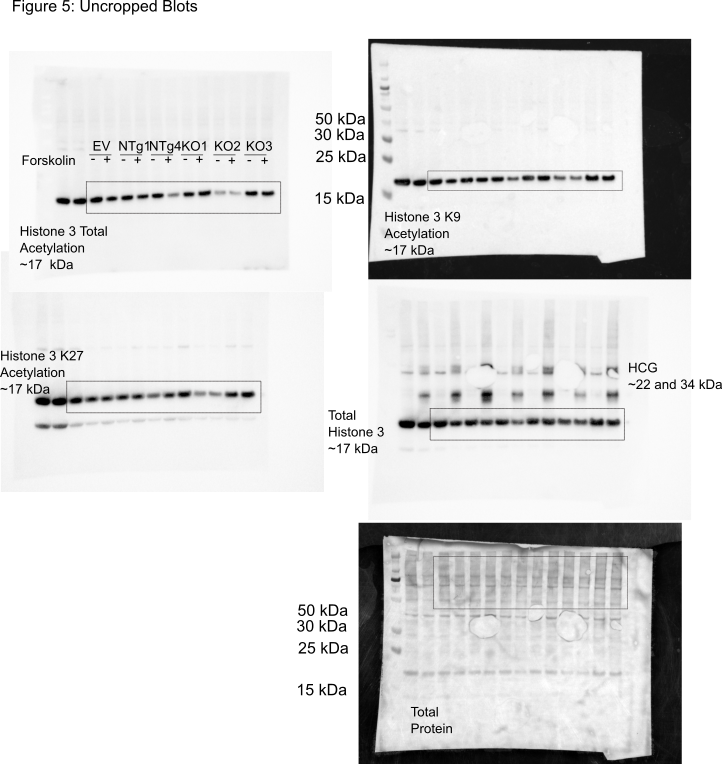

Supplement: Supplementary file 1 — Supplementary Figures. [file 41598_2023_34435_MOESM1_ESM.docx]
