# Supplementary material for: Mitochondrial citrate metabolism and efflux regulate BeWo differentiation
Source: Sci Rep. 2023 May 6;13:7387. doi: 10.1038/s41598-023-34435-x (PMC10164164; doi:10.1038/s41598-023-34435-x)
Supplement: Supplementary file 3 — Supplementary Legends. [file 41598_2023_34435_MOESM3_ESM.docx]

**Supplemental Table 1 Legend:**

Differential Gene Expression of CIC Knockout and Control cells treated with DMSO and Forskolin:

Sheet 1: Differential gene expression of DMSO treated CIC knockout cells compared to control using absolute fold change of 1.5, *p* adjusted <0.01.

Sheet 2: Differential gene expression of forskolin treated CIC knockout cells compared to control cells using absolute fold change of 1.5, *p* adjusted <0.01.
